# Novel Concepts in Psoriatic Arthritis Management: Can We Treat to Target?

**DOI:** 10.1007/s11926-018-0781-x

**Published:** 2018-09-18

**Authors:** Laura J. Tucker, Weiyu Ye, Laura C. Coates

**Affiliations:** 1 Nuffield Department of Orthopaedics, Rheumatology and Musculoskeletal Sciences, Botnar Research Centre, University of Oxford, Windmill Road, Oxford, OX3 7LD UK; 2 Oxford University Clinical Academic Graduate School, University of Oxford, Oxford, UK

**Keywords:** Psoriatic arthritis, Treat to target, Minimal disease activity, Remission, Tight control, Biomarkers

## Abstract

**Purpose of review:**

Psoriatic arthritis (PsA) is a chronic inflammatory spondyloarthritis that can cause progressive joint damage and irreversible disability. Advances in modern therapies, now mean a target of remission is an achievable goal in PsA. There is strong and consistent evidence that a treat-to-target (T2T) approach to PsA management results in better patient outcomes; however, the practicalities of incorporating this strategy into routine clinical practice remain a challenge. The heterogeneous nature of this condition and the need for validated outcome measures have to-date hampered consensus on a definition of remission. This review aims to summarise the current T2T research landscape in PsA and highlight potential roles for biomarkers and imaging advances in revolutionising the T2T concept.

**Recent findings:**

There is a growing body of evidence to support the implementation of a T2T strategy, using a pre-defined target in PsA management, with significant benefits in disease outcome, physical function and quality of life.

**Summary:**

Whilst remission is the ultimately goal for PsA patients and their clinicians, further comparative studies of different treatment targets are needed to establish a widely acceptable definition of remission.

## Introduction

Psoriatic arthritis (PsA) is a chronic, progressive inflammatory spondyloarthritis, affecting up to 30% of those with psoriasis [1]. It is a multifaceted condition, encompassing a variety of clinical phenotypes, including arthritis, enthesitis, dactylitis, psoriasis and axial disease. For this reason, the term psoriatic disease may be more appropriate. PsA has a similar impact on function and quality of life to that of rheumatoid arthritis (RA) [2], with 20% of PsA patients developing irreversible joint deformities and permanent loss of function [3]. Despite a growing body of evidence to support early diagnosis and treatment of PsA [4–7], disease heterogeneity presents a major obstacle to achieving this. Improved knowledge of the disease pathogenesis has enabled the rapid development of effective targeted therapies, which have significantly changed the scope of PsA treatment and led to important treatment advances. This now means that levels of disease control previously thought to be impossible in PsA, such as remission, have become realistic goals of therapy.

The concept of treat-to-target (T2T) was originally developed for chronic diseases, such as diabetes mellitus, hypertension and hypercholesterolaemia and was found to improve clinical outcomes [8–11]. More recently, we have seen a similar paradigm shift in the field of rheumatology [12–14], with the adoption of a T2T approach in rheumatoid arthritis (RA) revolutionising patient outcomes [12]. This has been translated into PsA with the recent publication of the TICOPA (TIght COntrol of Psoriatic Arthritis) trial. This was the first study to provide evidence that T2T in PsA improves patient outcomes compared to standard care [15••]. However, given the highly heterogeneous nature of PsA, there is poor agreement as to which target of response should be utilised, and the practicalities of incorporating T2T into routine clinical practice remain a challenge. This review aims to summarise the current T2T research landscape and address the exciting role biomarkers, and recent imaging advances have to play in the delivery of the T2T concept in PsA.

## The Importance of the T2T Concept in PsA

T2T is a strategic approach to guide treatment towards a pre-specified target. Validated measures are utilised to regularly and objectively assess disease activity, and therapy is adjusted accordingly to meet this target. This novel strategy is fast becoming the standard of care in rheumatology. The concept of T2T in RA evolved based on evidence to support the association between inflammation and joint damage in RA [16]. The TIght COntrol of Rheumatoid Arthritis (TICORA) study showed that a T2T approach in RA led to improvements in disease activity, radiographic progression, physical function and quality of life [12]. A number of other RA studies, including the randomised controlled trials (RCTs) CAMERA and STREAM, have mirrored these findings, cementing T2T’s place in the management of RA [17–23].

Interest has now extended to the PsA and spondyloarthritis (SpA) arena. Over the last 5 years, sufficient evidence has accumulated that inflammation is pivotal to progressive joint damage in PsA [24, 25], with pain and swelling in a joint predicting radiographic outcomes [26]. Thus, the ultimate aims of T2T in PsA are to eliminate joint inflammation and prevent structural joint damage, thereby maximising health-related quality of life and function, whilst minimising disability. Indeed, observational studies have suggested that early diagnosis and treatment of PsA are associated with better clinical, radiographic and functional outcomes [4–7].

The TICOPA study was the first RCT to confirm benefit from a T2T strategy in PsA [15••]. This multi-centre, open-label RCT used the seven-component minimal disease activity (MDA) criteria as the pre-defined target. Patients were deemed to be in a state of MDA if they met five of the seven criteria tender joint count ≤ 1, swollen joint count ≤ 1, Psoriasis Area and Severity Index (PASI) ≤ 1 or body surface area (BSA) ≤ 3, patient pain visual analogue score (VAS) ≤ 15, patient global disease activity ≤ 20, health assessment questionnaire (HAQ) ≤ 0.5, tender entheseal points ≤ 1 [27]. DMARD-naïve adult patients with recent onset PsA were randomised 1:1 to receive either tight control or a standard, non-steered treatment approach, for 48 weeks. Those in the tight control group were reviewed every 4 weeks by a research rheumatologist with escalation of treatment using an algorithm if MDA was not achieved. Those in the standard care arm were reviewed every 12 weeks by their usual rheumatologist and received standard therapy, without the use of an algorithm. The primary outcome was the proportion of patients achieving an American College of Rheumatology 20% (ACR20) response at week 48. The odds of achieving ACR20 (OR 1.91, *p* = 0.0392), as well as ACR50, ACR70 and PASI75, were significantly higher in the tight control group. Improvements in patient-reported outcomes, such as physical function and quality of life, were seen with tight control. An increased rate of adverse events and serious adverse events was noted in the tight control group. This may be secondary to the use of combination therapies and rapid treatment escalation, or may partly reflect reporting bias owing to more frequent clinical reviews [15••]. There remains a need to determine the longer-term impact of T2T. Furthermore, the successful clinical translation of TICOPA requires an agreement on a pre-defined, quantifiable target for PsA.

## Defining the Treatment Target

The heterogeneity of PsA and paucity of validated outcome measures has hampered consensus on the most appropriate target to use in a T2T approach to PsA. Given that biological and targeted synthetic DMARDs can be highly effective in PsA, some have advocated remission as the ideal treatment target [28••, 29••, 30••]. The concept of remission is the complete absence of disease activity, including skin, and implies adequate disease control such that sequelae are avoided and quality of life maintained [31]. However, despite numerous attempts to define remission in PsA, a universally accepted definition remains elusive.

### Comparison of Different Composite Measures to Assess Disease Activity

The RA-derived ACR response criteria and 28-joint Disease Activity Score (DAS28) are severely limited in their ability to assess all disease domains [31, 32] and the use of reduced joint counts will result in misclassification of patients with oligo- and mono-articular disease, especially if disease affects ankles, feet or distal interphalangeal joints [33]. Subsequently, these measures neglect significant numbers of patients with active psoriatic disease. Despite this, a recent physician survey undertaken by the GRAPPA-OMERACT group found that 19.5% of healthcare professionals still use DAS28 as a measure of remission or low disease activity (LDA) in PsA [28••].

In 2017, Mease et al. reviewed the available literature on PsA treatment targets, including MDA [34]. They concluded that given the multifaceted nature of PsA, any target should reflect this and objectively assess five key disease domains: synovitis, dactylitis, enthesitis, spondylitis and psoriasis/ nail disease [34]. The two most commonly discussed composite measures in PsA, minimal disease activity (MDA) and Disease Activity in PSoriatic Arthritis (DAPSA), encompass the full 66/68-joint counts. However, the DAPSA assesses only peripheral arthritis whereas the MDA criteria assess relevant clinical outcomes across several domains. These measures are being increasingly used in clinical and research practice [15••, 25, 27, 35–37].

The use of cut-off points of these scores as therapeutic targets has been shown to be achievable and acceptable goals. Remission and low disease activity criteria have been defined and validated for DAPSA, with a cut-off value for remission of ≤ 4, and values between > 4 and ≤ 14, > 14 and ≤ 28 and > 28 for low, moderate and high disease activity respectively [35]. Whilst DAPSA can be undertaken quickly, making it appropriate for day-to-day clinical practice, it only assesses articular disease and thereby provides only a very limited assessment of the psoriatic disease spectrum. In contrast, MDA includes assessment of the wider range of psoriatic disease and can be easily incorporated into clinical practice with simple measures of enthesitis and psoriasis in addition to peripheral arthritis. MDA is achieved when any five of the seven criteria are fulfilled, whilst patients are said to have very low disease activity (VLDA), which could represent a state of remission, if all seven criteria are fulfilled [27]. However, the MDA composite measure is only a binary tool and cannot quantify the degree of disease activity or response on a continuous scale.

In addition to the target being feasible to assess in clinical practice, it must also be attainable. Several studies have shown that a state of MDA is achievable in most PsA patients treated with anti-TNF therapy [38, 39]. A recent Spanish cross-sectional multi-centre study found that 60% of Spanish PsA patients achieve MDA in routine clinical practice, with a significantly lower impact of disease observed in those patients who achieved MDA [40]. Whilst similar rates of low disease activity using several composite measures were observed over a 10-year period in a recently published Norwegian study, no significant improvement in patient-reported outcomes (PROs) was observed over the same timeframe. However, in this study, no comparison was made between PROs in those patients achieving remission versus those who did not [41].

Unsurprisingly, multiple studies of composite indices have found that remission rate estimates differ between available composite measures, and few data exist to identify which strategy is optimal given the risks and benefits of aggressively targeting therapy [42]. Using a dataset of 250 PsA patients who had been deemed to have quiescent disease by their rheumatologist, van Mens et al. compared these composite scores and found that whilst there was significant overlap between them, they differed in their allowance of residual disease. The absence of skin disease in the DAPSA measure, meant some patients who were deemed to be in ‘remission’, in fact had a reduced quality of life due to residual skin disease [43]. On further assessment, Van Mens et al. also demonstrated that clinicians accepted higher levels of disease activity compared to the MDA criteria. They found that one third of PsA patients deemed to be in an acceptable disease state according to their treating rheumatologists did not meet the MDA criteria, with a significant impact on quality of life, function, mental health and work productivity [44]. This study highlights the discrepancy between current clinical practice and target driven assessment and should compel clinicians to incorporate simple assessments, such as MDA or DAPSA, into routine clinical practice.

### Patient Perspective on Disease Activity and Defining Remission

It is important to remember that discrepancies between patient and physician perceived disease activity may occur. For many patients, remission of inflammation may not equate to complete absence of all symptoms and may not reflect the residual impact of disease on quality of life and function. In a study of 565 patients with PsA, Eder et al. assessed the discordance between patient and physician global assessments and found that patients tended to score higher in their disease assessment than physicians [45]. Similarly, the recent prospective, multi-centre NOR-DMARD study showed differences in patient and physician assessments of disease activity and tender and swollen joint counts, with patients placing a greater emphasis on pain. They concluded that such discordance may reduce the chance of achieving remission in PsA [46].

Importantly, patient definitions of remission often focus on the impact their disease has on their quality of life and function, areas that are not always sufficiently covered in many self-reported outcome measures [47]. For example, in a RA study undertaken by van Tuyl et al., they found that patients with RA consider remission to be more of a feeling of return to normality than a reduction in symptoms [48]. There is therefore a disconnect, where patients may not perceive themselves as being in remission, which may not be related to ongoing disease activity, but due to joint damage, comorbidities or chronic pain.

In addition, patients’ expectations will vary between individuals and be heavily influenced by the exact disease manifestations. Lubrano et al. recently demonstrated that up to a quarter of patients deemed to be in remission or LDA using DAPSA and MDA have residual disease activity, mainly in the form of skin disease [49]. It could be argued that rather than aiming for disease remission or LDA, identifying a ‘target to treat’, in other words, targeting the aspect of disease most significant to each individual patient may be a more appropriate strategy.

## Current T2T Recommendations in PsA

The T2T strategy in PsA is supported by the international T2T task force, European League Against Rheumatism (EULAR) and Group for Research and Assessment of Psoriasis and Psoriatic Arthritis/Outcome Measures in Rheumatology (GRAPPA-OMERACT) [28••, 29••, 30••, 50••]. These international recommendations highlight the uncertainty surrounding definitions of remission and the ideal composite measure. However, the general consensus amongst clinicians and patients alike is that a state of remission should be the primary treatment target, with LDA/MDA used as an alternative in certain circumstances, such as long-standing disease, fixed joint deformities, comorbidities and patient goals [28••, 29••, 30••]. Unless there are clear risks that prevent treatment escalation, higher disease activity states are unacceptable.

In a recent patient survey, the GRAPPA-OMERACT group found that 57% of healthcare professionals and 56% of patients with PsA believed that remission should be the optimal treatment target, with LDA or MDA used as an alternative[28••]. Furthermore, in 2017, the international T2T task force found that the overwhelming majority of participants (88.9%) comprising patient representatives, non-healthcare professionals and rheumatology and dermatology healthcare professionals, approved the recommendation of remission/ inactive disease as the primary treatment target [30••]. Some participants stated that progressive joint damage may potentially occur even in the context of LDA [24, 25], whilst the concept of remission implied a cure-like state with no further joint damage [30••]. Crucially though, it is not known if targeting remission does improve outcomes for patients and what potential risks this may introduce with more aggressive drug therapy.

## Implementation of T2T in Clinical Practice

Despite the availability of PsA specific outcomes measures and international recommendations encouraging clinicians to use the T2T strategy as their standard approach to PsA management, it is yet to receive wide implementation. A GRAPPA-OMERACT physician survey found that only 56% of healthcare professionals are using T2T in clinical practice when managing these PsA patients, with MDA being the most popular target used (32%). This highlights the lag in translating best practice from academia into day-to-day clinical practice.

Amongst clinicians, the general consensus is that the treatment target should be individualised, taking into account patient comorbidities and patient preference for disease activity, whilst also incorporating patients’ concerns over perceived drug safety, cultural beliefs and drug-related risks. Although tight control of psoriatic disease has a number of associated benefits, it is not without risks. The TICOPA study demonstrated that more intensive therapy associated with tight control may result in greater drug-related side effects [15••]. Pursuing a target of remission may therefore not be appropriate in certain settings, such as in patients predisposed to recurrent infections. It is also recognised by many that strict remission may be difficult to achieve and maintain in many patients, particularly in those with long-standing disease and subsequent damage and functional impairment, as well as those with significant comorbidities, such as obesity [51]. A recent study found that 42% of patients with PsA have ≥ 3 comorbidities [52], highlighting this as a potential hurdle in achieving disease remission. Therefore, in these circumstances, MDA, ‘near-remission’ or LDA states may be sufficient [31].

The overarching principles of the international T2T task force state that the treatment target must be based on a shared decision between clinicians and patients, as pursuit of remission may not be appropriate for all patients [30••]. However, a patient survey undertaken by GRAPPA-OMERACT found that the majority (61%) of patients with PsA had not discussed personal management goals with their rheumatologists and one in five patients wanted their rheumatologists to spend more time listening to their concerns [28••]. Furthermore, patient education in PsA has been shown to be suboptimal, with PsA patients being less empowered compared to their RA counterparts [53]. Ultimately, involvement of patients in discussions regarding their proposed treatment strategy will be pivotal in improving patient satisfaction and implementing T2T in routine clinical practice.

Furthermore, in order to optimally manage patients with PsA, all aspects of psoriatic disease, including the extent and severity of skin disease, need to be considered and assessed using validated instruments. This can be challenging in time-pressured clinics. Whilst some composite measures are more complex and time consuming, both DAPSA (alongside validated measures of enthesitis and psoriasis) and the MDA criteria are quicker to undertake and easy to calculate in the clinic. The GRAPPA-OMERACT physician survey found that most clinicians believed that the ideal composite measure should balance accuracy against the time taken to complete an assessment, with a duration of < 10 min or ideally < 5 min believed to be reasonable [28••].

At present, MDA criteria are the most representative composite measure of psoriatic disease, whilst also being feasible to perform in clinical practice. Patient-reported outcomes can be recorded by the patient whilst in the waiting room, prior to their clinic appointment, with MDA taking patients approximately 5–10 min to complete. Currently, < 25% of patients are asked to complete any questionnaires prior to their appointments, with 91% being willing to do so if asked [28••]. The MDA is a relatively short assessment, and with regular use by clinicians, clinical assessment of various disease domains can take 5–10 min [54].

Clearly, to enable the contemporaneous adjustment of treatment, assessment of disease activity cannot be a discrete event but should instead be undertaken regularly. However, there is a lack of data on the best time interval for monitoring patients. Patients in the tight control arm in the TICOPA study were followed up at 4-week intervals [15••]. In real-life clinical practice, this may not be feasible nor necessary for those patients who have recently changed therapy or who have achieved their pre-defined treatment target. The 2015 EULAR recommendations for PsA management recommend ‘regular monitoring’ of patients with PsA and suggest monthly to 3-month monitoring in patients with active disease [29••]. Gladman et al. recently published recommendations on applying T2T in Canadian clinical practice and recommended that patients with active disease should be reviewed and medications adjusted every 3 months in clinic, with those in whom therapeutic targets have been reached, being reviewed every 6–12 months [55]. There remains a need to determine whether a reduction in the frequency of rheumatology visits will dilute the beneficial effects of T2T.

## Cost-Effectiveness of a T2T Strategy

The severity and chronicity of PsA translate into high direct and indirect healthcare costs [56, 57]. Such costs range from £11 to £20,782 per patient per year, with a mean annual healthcare cost of £1446 per biologic-naïve PsA patient in the UK [58]. These costs correspond to studies assessing the financial implications of PsA in other European countries [56, 59]; however, higher direct healthcare costs are associated with PsA in the United States (USA). One US study utilising data from 2011 to 2012, estimated an average annual direct cost of $19,282 per patient per year, which when adjusted to exclude bDMARDS, equated to an annual cost of $5258 per patient [60].

Growing general healthcare costs alongside fixed government funding is putting ever greater emphasis on delivering clinically effective healthcare at the least possible cost. T2T is more established in RA and whilst many commissioners support a T2T strategy in early RA, there is limited enthusiasm in established disease and therefore, there has been a call for stronger evidence on efficacy and cost-effectiveness of T2T strategies.

Whilst a tight control strategy has been shown to be clinically effective [15••], cost-effectiveness analyses of tight control versus standard care in the TICOPA study concluded that tight control was not cost-effective in this trial [15••]. Whilst little difference was found in quality-adjusted life years (QALYs) gained between tight control and standard control [61], this was only measured over 48 weeks. Sensitivity analysis incorporating decreasing drug prices with the increased use of biosimilars, and reducing rheumatology visits as patients are established on maintenance therapy, suggested improved cost-effectiveness in acceptable ranges. Further, since T2T conferred greater benefit for polyarthritis patients [15••, 61], its cost-effectiveness may be leveraged through a more targeted application. Ultimately, longer-term follow-up studies and data from real-life implementation of T2T in clinical practice will be invaluable in assessing cost-effectiveness and evaluating whether the additional clinical benefits of tight control persist and translate into improved QALYs.

## Are There Roles for Immunological and Imaging Markers in Defining Remission?

### Biomarkers of Disease Activity

Despite significant advances in molecular ‘omic’ technologies in the last decade, as yet, there are no validated biomarkers for diagnosis of psoriatic disease, prediction of treatment response or identification of those in remission. Whilst standard acute phase reactants, such as C-reactive protein (CRP) and erythrocyte sedimentation rate (ESR), serve as markers of disease activity in PsA, they are not specific to psoriatic disease. There is compelling evidence that the IL-17/IL-23 pathway is central to the pathogenesis of both psoriasis and PsA [62–68]. Menon et al. demonstrated that synovial fluid from patients with PsA contains increased percentages of Th17 and IL-17 CD8+ T cells (Tc17) compared to peripheral blood, and in contrast to synovial fluid from patients with RA, in which Th17, but not Tc17, cells are increased compared to peripheral blood. Furthermore, they found that the percentage of Tc17 cells in synovial fluid of patients with PsA positively correlated with disease activity markers and radiographic erosion status after 2 years [63].

A number of serum-soluble bone and cartilage-turnover markers in PsA and psoriatic spondyloarthritis has been identified as potential biomarkers of disease. These include matrix-metalloproteinase (MMP)-3, Dickkopf (DKK)-1, macrophage colony-stimulating factors (M-CSF) and osteoprotegerin (OPG) [69, 70]. A recent prospective cross-sectional comparative study confirmed that these four biomarkers are associated with PsA. MMP-3 and M-CSF were found to be biomarkers for the presence of psoriasis in psoriatic disease and could potentially be used to screen for PsA in patients with psoriasis. Concentrations of DKK-1 and OPG could differentiate between those PsA patients with and without axial arthritis and could therefore be used to screen for the presence of axial disease in PsA and in addition, help differentiate psoriatic spondyloarthritis from ankylosing spondylitis [71]. Longitudinal studies are needed to validate these biomarkers against disease activity.

Advances in our understanding of PsA pathobiology will help to identify new biomarkers of psoriatic disease, define immunological remission in a T2T strategy and allow clinicians to practise more effective and personalised medicine.

### Using Imaging to Monitor Disease Activity and Define Remission

Modern imaging techniques, such as ultrasound (US) and magnetic resonance imaging (MRI), have advanced our understanding of the pathogenesis of the various PsA phenotypes and have proven useful adjuncts in the diagnosis of psoriatic disease, including subclinical disease [72]. Both imaging modalities enable early detection of inflammatory changes within articular and periarticular structures and evaluation of the extent of structural damage [72–77]. They have been shown to be more sensitive in identifying certain PsA pathologies than clinical examination [75]. US-detected synovitis and enthesitis are associated with long-term radiographic progression and poor outcomes [78, 79]. Whilst these imaging modalities are predominantly used to diagnose psoriatic disease, they are being increasingly used in both clinical and research practice to objectively measure treatment response. Indeed, EULAR recommendations support this practice [80].

These highly sensitive imaging techniques have the potential to improve PsA management; however, as yet, their exact roles in defining remission in PsA and reflecting subclinical disease activity are not known. This has been more extensively evaluated in RA, where the ARCTIC study compared two tight-control treatment strategies for early RA, with the aim of determining whether a treatment strategy based on structured ultrasound assessment would lead to improved outcomes in RA. One hundred twenty-two patients were randomised to an ultrasound tight control strategy targeting clinical and imaging remission and 116 patients to a conventional tight control strategy targeting clinical remission. Despite more aggressive treatment in the ultrasound group, no differences in joint swelling, clinical remission or inhibition of radiographic joint damage were observed between both groups [81•]. Similarly, the TaSER study showed that an US driven T2T strategy led to more intensive therapy, but was not associated with significantly better clinical or imaging outcomes [82•]. Overall, both studies concluded that implementation of US in routine follow-up of patients with early RA was not justified, with the ARCTIC study also suggesting that the lack of gain in benefits and the increased costs, time consumption and use of bDMARDs associated with the ultrasound tight control regimen would yield negative cost-benefit ratios [81•]. These study outcomes are somewhat surprising, given the growing body of evidence to suggest that subclinical inflammation is associated with radiographic progression and disease flares [78, 79, 83–85]. Further studies are needed to establish the correlation between sonographic and histologically confirmed inflammation and assess the efficacy of US within the sphere of PsA. At present, there is insufficient evidence to support the routine use of US assessment as part of an enhanced T2T strategy in newly diagnosed inflammatory arthritis.

## Conclusion

There is growing evidence to support the T2T concept in PsA. With the advent of modern therapy, achieving a disease state that avoids progression, prevents joint damage and optimises long-term function is now a realistic goal. However, there remains a need to define remission in a way that is acceptable to patients, clinicians and researchers alike. Ultimately, shared decision-making between the patient and rheumatologist will be pivotal to improving patient satisfaction and management of this long-term condition.

An increased understanding of the pathways that drive the pathogenesis of PsA, through advances in molecular ‘omic’ technologies will be key in identifying specific biomarkers and defining immunological remission in psoriatic disease. Whilst modern imaging techniques have proven useful adjuncts in the diagnosis of psoriatic disease, it is not yet clear whether radiological remission provides any added benefit beyond clinical composite measures.

T2T is an exciting and rapidly evolving research field. Future comparative studies will need to establish the efficacy of novel treatment targets, assess whether meeting more stringent targets, such as remission, provides additional clinical benefit and determine if longer-term improvements translate into cost-effectiveness.
